# Emotion Expression in Modern Literary Appreciation: An Emotion-Based Analysis

**DOI:** 10.3389/fpsyg.2022.923482

**Published:** 2022-06-17

**Authors:** Jingxia Li

**Affiliations:** ^1^Shanxi University, Taiyuan, China; ^2^Shanxi Vocational University of Engineering Science and Technology, Jinzhong, China

**Keywords:** modern literary appreciation, emotion expression, emotion classification, emotion analysis, machine learning

## Abstract

**Background:**

Modern literary appreciation seems to be reading literary works phenomenally. In fact, appreciation is not a general reading, which has an important difference from general reading. It is the identification and appreciation of literary works and a complex spiritual activity for people to feel, understand, and imagine literary and artistic works. At the same time, literary appreciation is also a cognitive activity, an aesthetic activity, and a re-creation activity.

**Method:**

In this paper, the machine learning algorithm was creatively used to classify the emotions of figures in modern literary works, to analyze the emotions of the figures that the writer wanted to depict in modern literary works.

**Results:**

Experimental results verify the accuracy of the emotion classification method through experiments, which is helpful for us to better understand the emotion expression in modern literary works.

## Introduction: Common Perspectives on Appreciating Modern Literature

The appreciation and learning of modern literary works is a complicated and multifaceted process (Qin, 2018). It is mainly a re-creative imagination along with the track of the works' images and hints, imagining their state and fate according to the characters and scenes described in the works, and then reconstructing aesthetic images in the mind (Lian, 2014; Mak and Willems, 2019). It can be seen that the appreciation of modern literary works is internal, relying on a word to have a deep understanding of artistic images. In the process of appreciation of modern literary works, readers can better understand and feel the writer's thoughts and feelings and have a deeper reading experience (Magulod, 2018; Sunaryo, 2018).

The image of modern literary works mainly refers to the vivid and specific life picture created by literary works that can stimulate people's thoughts and emotions and usually refers to the figures portrayed by modern literary works and their spiritual outlook and personality characteristics (Suhendi, 2017; Li, 2018; Wen and Piao, 2022). The analysis of the characters in a literary work is essentially the appreciation of the image of literary works (Yandell, 2017). In the appreciation of modern literary works, on the one hand, we should pay attention to reveal the typical meaning of the figures, on the other hand, we should focus on analyzing the characteristics of the figures.

When appreciating modern literary works, we should pay attention to two problems. First, the image of works should be appreciated from the perspectives of words, actions, and forms of the characters in the works, rather than blindly labeling the characters or imposing on the characters what the characters do not have. Second, it is necessary to make an effective analysis of the personality of the characters in the works. Personality can be reflected not only in general terms but also in the details of the whole work. When appreciating the language of literary works, we should understand the image, typicality, and emotional characteristics of literary language (Guy et al., 2018). Then, it is necessary to realize that vividness is the highest level of literary language. The appreciation of modern literary language mainly includes the following aspects: (i) to analyze the characteristics of language, (ii) to understand the linguistic style of literary works, and (iii) to discriminate the rhetoric and expression effect applied in literary works. The appreciation of language mainly includes the following aspects. First, appreciating the language of works from a rhetorical point of view. Second, appreciating the language of works from the perspective of expression methods. Common expressions in modern literary works include narration, description, discussion, lyricism, and explanation, etc. Clear expressions can make the language of articles flexible and diverse. Third, appreciating the language of works from the perspective of words. Good modern literary works are characterized by colorful languages. Therefore, we should pay more attention to the use of words when appreciating modern literary works.

Conception is the writer's general idea of writing from content to form, which can be considered in terms of idea, material selection, style, artistic conception, and structure (Atasoy, 2021; Fowler, 2021). There are three main types of conceptual approaches in literary works: vertical conception (arranging materials in chronological order), horizontal (selecting several aspects of a thing or several contents of a problem), and vertical and horizontal (combining vertical and horizontal methods when selecting materials). While the well-constructed conception of literary works includes two levels, one is the well-constructed ideas and the other is the well-constructed structure.

The contribution of this paper is that a machine learning-based emotion classification method is proposed for the recognition of emotions of figures in modern literary works. The rest of the paper is structured as follows. In Section Emotion Expression in Modern Literature Appreciation, the emotion expression in modern literature appreciation is studied. The analysis method and experimental results are reported in Section Method and Results. Section Discussion gives the discussion.

## Emotion Expression in Modern Literature Appreciation

The process of appreciating literary works is a process of obtaining effective information, experiencing an aesthetic feeling, interpreting the thoughts and emotions of the works, and tasting the personality expression skills and language art of the characters in the works (Kuijpers and Hakemulder, 2018). When writers create literary works, they systematically and pertinently depict the appearance, psychology, and behavior of the characters in literary works by using various decorative techniques, such as description, rhetoric, and grammar (De Vita et al., 2021). Therefore, it is very important to correctly grasp the language in literary works appreciation to improve the level of works appreciation. To improve the ability and level of appreciation of literary works, we should have a deep understanding of the language in appreciation of literary works, correctly interpret the ideological connotation of works, and experience the language beauty and ideological quality of works.

The emotional expression of literary language is an important form of expression in the carrier of literature, which contains both language art and emotional art (Parente et al., 2020). It can be said that emotional expression is the link between literature and people. It is quite common to use literary language to express emotions in human activities. Writers convey their experience and feelings of certain external things to others, and others also achieve the effect of experience and feelings through such emotional transmission, which reflects the significance of emotional expression. In this process, human beings experience, perceive, recognize, understand, and summarize things with an aesthetic attitude, so the emotional expression in writers is the key factor of literary language expression. In the process of appreciating literary works, the works themselves attract readers with the expression and resonance of emotion and soul. From the connotation of the expression of emotion in literary language, this paper discussed the main forms of the expression of emotion in literary language and analyzed the expression of negative emotion in the literary language in detail.

### Connotation of Emotional Expression in Literary Language

Literary writers are different from painters and photographers because they can quickly material in real life, such as moving trains, high-rise buildings, childlike eyes, and so on, while literary writers need to express through words, which is not a sensory art. In the process of writing, writers cannot describe contents directly or mechanically but need to express emotions; only in this way can things be interpreted better. The expression of emotion is very obvious in the lyric works, while the expression of emotion is another form in the documentary or narrative works. No matter which literary language needs a certain internal drive. Driven by this emotion, readers can quickly get into the emotional environment in the process of reading. At the same time, writers can really arouse the desire to create and write moving works.

The elements of the expression of emotion in literary language are mainly evolved from the semiotic system. Human language has practical and aesthetic functions. As a way of expression, literary language also has the aesthetic feeling and aesthetic taste of language form. In the process of expressing works, writers need to fully express their personal experiences and emotion in the form of symbols, and specific sentences or words cannot only make the expression of emotion more wonderful but also full of the emotion of symbols. The writer's spiritual feeling and emotional experience in the literary language is often closer to life and at the same time, has a certain appeal to reach the resonance between readers and writers. Therefore, in the process of reading, we should pay attention to the writer's expression of language feelings, so as to understand the writer's description of the artistic conception of the work.

### The Main Form of Literary Language Emotion Expression

The phonetic variation of literary language is mainly to better convey the writer's unique understanding of life and aesthetic feelings, one of which is the writer's emotional experience of life. At the same time, readers can get more aesthetic pleasure and emotional experience when they appreciate literary works. The phonetic variation of literary language is mainly reflected in tone, rhythm, and so on. In modern poetry creation, the tonal harmony is often required, which can produce a kind of cadence harmony, resulting in a kind of pleasing and moving rhythm beauty.

The sememe contrast in the emotion of literary language is a common way of literary expression. It depends on the context of different figures of speech and word order, resulting in misalignment between literal, extended, and meaning. These misalignment forms create a three-dimensional space between the literal and the others, and this space is mainly for readers to expand their emotions and thoughts through the rich content of works, so as to enhance the liveliness, implication, ideological, and emotional nature of literary language, and then readers can be fully imagined and enlightened in the process of understanding. Thus, the change of sememe can truly reflect the change of thoughts and emotions. The larger the space of emotional thoughts, the richer the information loaded by literary language.

The combination and collocation of words are generally established by convention, with certain stability and standardization. However, in literary works, the combination and collocation of words often go beyond the convention due to the need of expressing feelings and thoughts. Such combination and collocation usually follow emotional logic rather than rational logic. This kind of transcendence can reflect the unpredictable, complex inner world of people, giving readers a broad space for emotional thinking and image thinking. In this space, readers can run freely and appreciate the connotation of different emotions and thoughts from different levels.

Literary language cannot accurately express thoughts and feelings without various contexts created by the writer, because the occurrence of human thoughts and feelings is always inseparable from specific situations. Literary language is also in the specific time, space, and logical environment to show their specific connotation. It is widely known that context refers to the actual environment in which a language is used, and context is different in scope and form. Integrated context refers to the specific social and natural environment in which language is expressed, which is easy to be ignored by people, while scattered context refers to the speech environment formed by the context of written language or speaking language, which is easy to attract people's attention. Since the use of literary language is always used in a certain context and is affected and restricted by the specific context, the language in the literary has its literal sense, that is, the meaning from the dictionary. Moreover, it also has connotations generated in a specific context, such as literal, extended, and meaning.

### The Expression of Negative Emotions in Literary Language

There are many expressions of negative emotions in literary language, and what is elaborated here is the expression of hatred. In fact, people are living in the conflict, literature is also inseparable from the performance of the various conflicts and description, and conflict could initiation hatred, hatred, and describes how unique performance, fully show hatred in colorful form, suspends in front of the complex problems of the writer. At the same time, hatred is also a kind of compound emotion of human beings, and its composition factors are complex. From the perspective of writing practice, it is closely related to the occurrence of tragedy, and its elements should include disgust, anger, resentment, and so on. Hatred should not be the patent of the injured and the good, the show of hatred for the despicable, but more can reveal the cruelty, danger, and hardship of life.

### The Psychological Emotion of Figures in Literary Works

The psychological emotion of figures in literary works is an indispensable part. It is necessary to describe the psychological state, spiritual outlook, inner activities of figures in a certain environment, and express the thoughts of figures, so that readers can penetrate the appearance of figures and observe their inner world. In literary works, there are many ways to portray figures, which are as follows: portrait description, language description, action description, psychological emotion description, and so on. Among them, psychological emotion is very important. Wonderful description of the figures' psychological feelings cannot only reveal the figures' personality characteristics but also reflect the change of the figures' thoughts, which can promote the development of plots and deepen the theme of literary works. In addition, psychological emotions can also delicately, vividly, and truly show the mental process of figures in literary works, which can directly penetrate into the figures' hearts, reveal their inner world, and express their rich and complex thoughts and feelings.

## Method and Results

### Classification Process of Figures' Emotions in Literary Works Based on Extended Corpus

Literary works have not only artistic value but also have social significance. The figures in literary works not only promote the plot development of literary works and enrich the depth and breadth of literary works but also provide good research and thinking significance for the society by the times, characteristics, and ideological connotations contained in these figures. Therefore, the appreciation of figures in literary works has not only aesthetic significance but also has the practical significance and ideological connotation. In many cases, the deviation or confusion of people's understanding and grasp of the figures in literary works is mainly due to the lack of systematic methods of appreciation of characters in literary works. However, the figures in good literary works will leave a deep impression on us. The description of the figure's emotions can better shape the typical character of the figure, so that the image of the figure is fuller, which is not only conducive to the highlight of the theme but also conducive to the appreciation of literary works. This paper used a machine learning algorithm to analyze the emotions of the figures in literary works, which helped us to appreciate the meaning expressed by the writers in literary works in a more scientific way, so as to better understand the writers' intentions.

**Figure 1:**
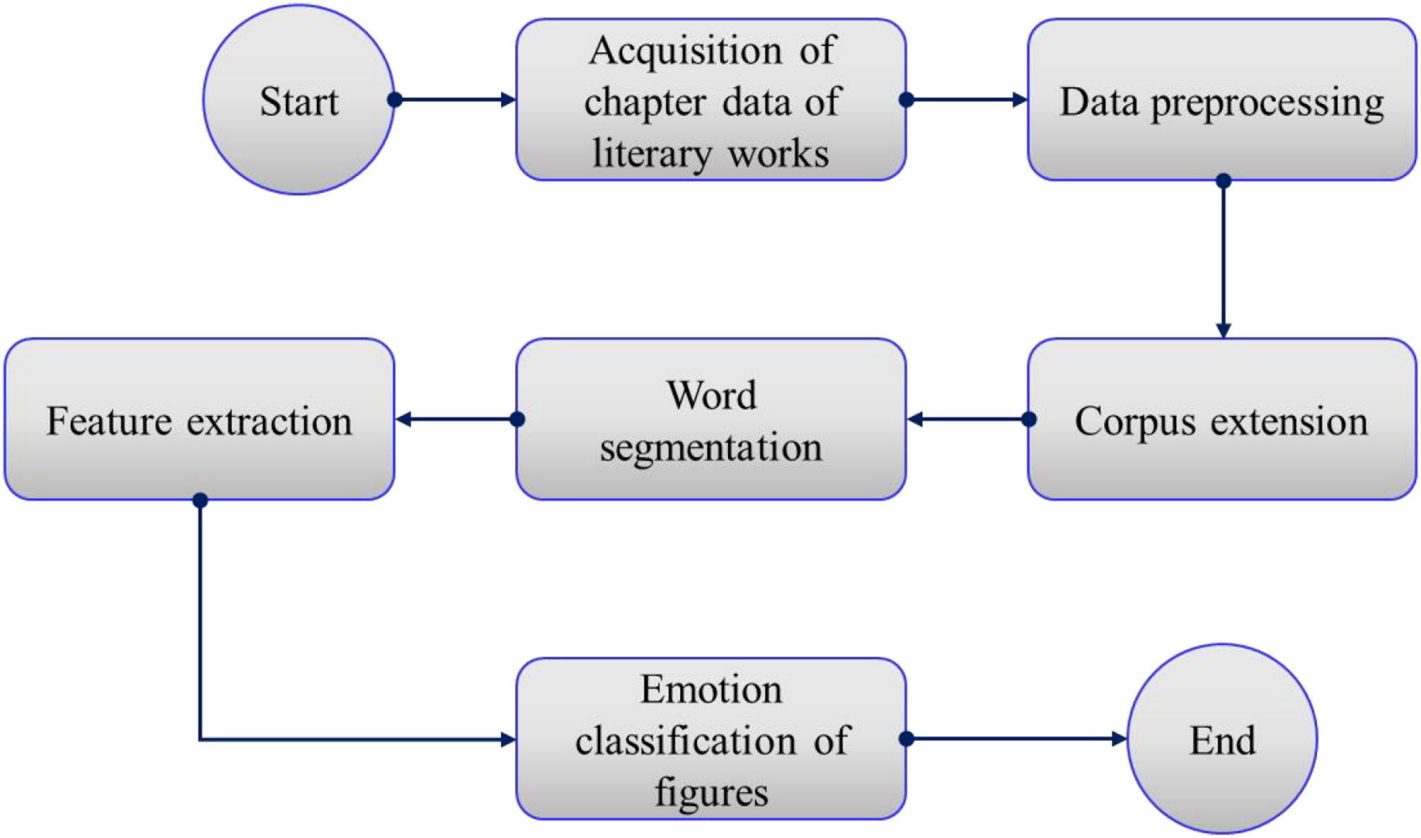
Classification process of figures' emotions in literary works based on the extended corpus.

In recent years, text sentiment analysis has gradually aroused the interest of industry and academia (Li et al., 2021). In the early work, the research focus was mainly on sentiment analysis based on positive and negative categories and analysis of positive, negative, and neutral aspects of emotional texts. However, binary classification sentiment analysis is difficult to fully express the complex inner world of human beings. It not only ignores the subtle emotional changes expressed by users but also fails to comprehensively cover the psychological state of users, which accelerates the demand for fine-grained sentiment analysis based on multiple classifications. Based on text length, sentiment analysis can be divided into three categories: word-level analysis, sentence-level analysis, and paper-level analysis. This study is based on the analysis of paper-level. However, in the field of Chinese text sentiment analysis, there are relatively few labeled datasets that fully meet the research needs and are authoritative enough to fully show the research results. At the same time, corpus expansion can remove part of the noise, alleviate the problem of feature sparsity to a certain extent, increase the semantically related space of text content, and form texts with similar semantics and different words, which can effectively improve the experimental effect of sentiment analysis technology. Therefore, after the extension of the corpus, this paper analyzed the emotions of the figures in literary works to corpus extension. The classification process of figures' emotions in literary works based on the extended corpus is shown in Figure 1.

The specific process is summarized as follows.

Step 1: Original corpus acquisition.

According to the research purpose of the emotions of figures shaped in literary works, all the information in the original corpus was constructed into corresponding strings, and then the strings were preliminarily matched according to the requirements to remove useless data information.

Step 2: Data preprocessing.

Literary works may contain some symbols, especially some modern literature, which may contain a typical network symbol of this period, and these labels have often useless information in the data. Therefore, data preprocessing needed to remove the part containing special symbols from the text content and the remaining part for text segmentation processing.

Step 3: Corpus extension.

After data preprocessing, it is necessary to detect negative words in corpus text, extend the corpus according to different emotional categories, and generate new corpus text. The new extended corpus was added to the original corpus set to form an extended corpus.

Step 4: Word segmentation.

The extended corpus was acquired and word segmentation was performed for the text content. Since there is no natural delimiter in Chinese, word segmentation is necessary for the text content. This paper used “Jieba” for Chinese word segmentation, which can customize the lexicon.

Step 5: Feature extraction.

We used the filter method for feature selection, extraction, sorting out the features in the Chinese text of literary chapters, and constructing feature sets as close as possible to the meaning of the original text content, and form feature vector space.

Step 6: Emotion classification.

The feature vector space in Step 5 was taken as the input, and the random forest (RF) algorithm was used to classify emotions and judge the emotional orientation of the extended corpus.

### Results and Analysis

To verify the accuracy and feasibility of the emotion classification algorithm based on the extended corpus, the Beijing Language and Culture University Corpus Center (BCC) corpus is used for verification (Cai et al., 2021). The scale of the Chinese corpus in BCC corpus is about 15 billion words, covering newspapers, literature, Weibo, science, ancient Chinese, and other fields. BCC corpus includes raw corpus, word segmentation corpus, part of speech tagging corpus, and syntactic tree. At present, part of speech tagging has been carried out on modern Chinese corpus. The extended corpus uses the People's Daily Chinese word corpus, Microsoft Research Asia Chinese word segmentation corpus, and WuDaoCorpus. The data in the corpus are mainly divided into three categories: positive, negative, and neutral. There are many positive emotion data in the corpus, while negative emotion data and neutral emotion occupy a small part of the dataset.

**Figure 2:**
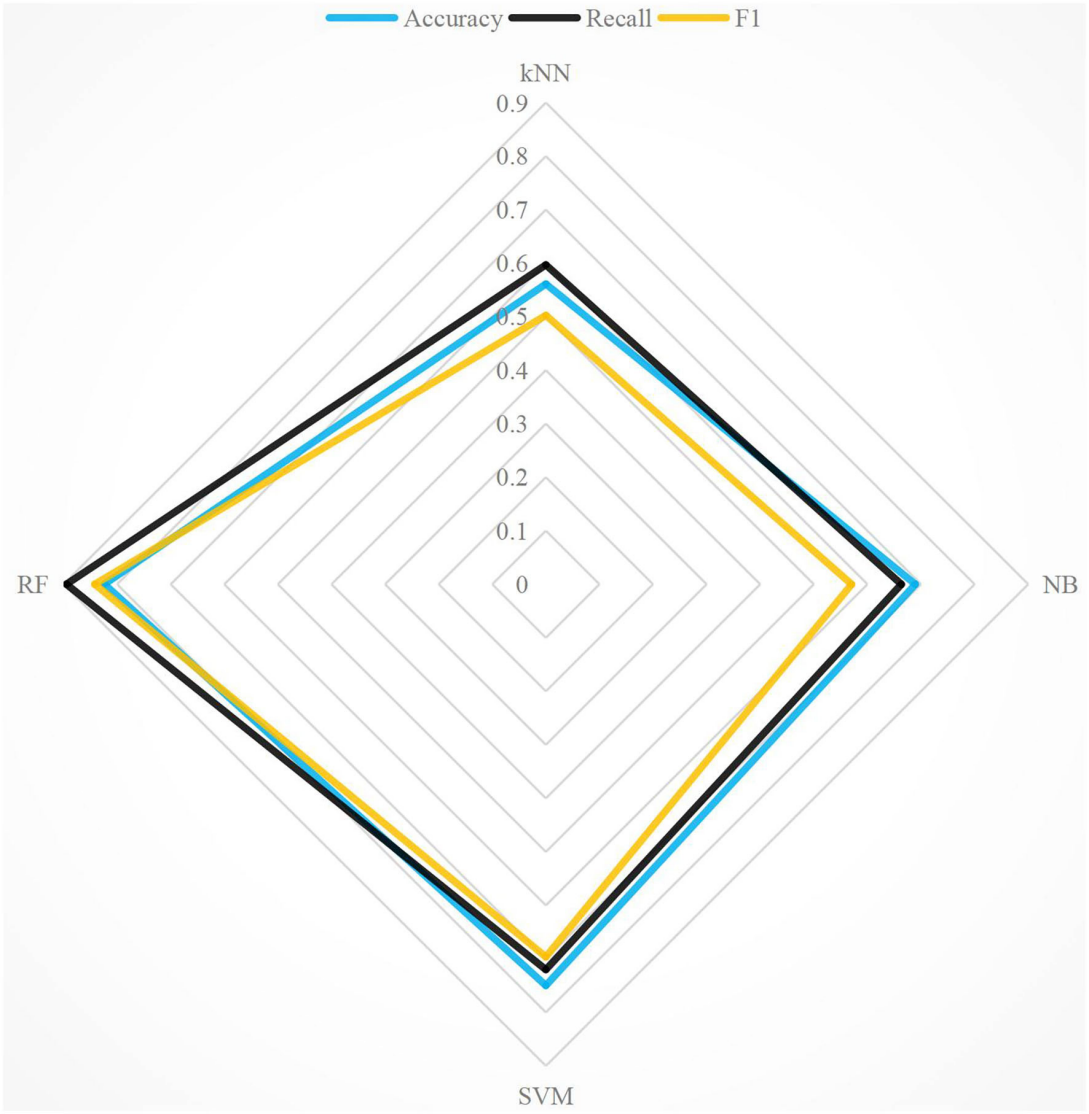
Comparison of evaluation metrics with four methods under Beijing Language and Culture University Corpus Center (BCC) corpus.

**Figure 3:**
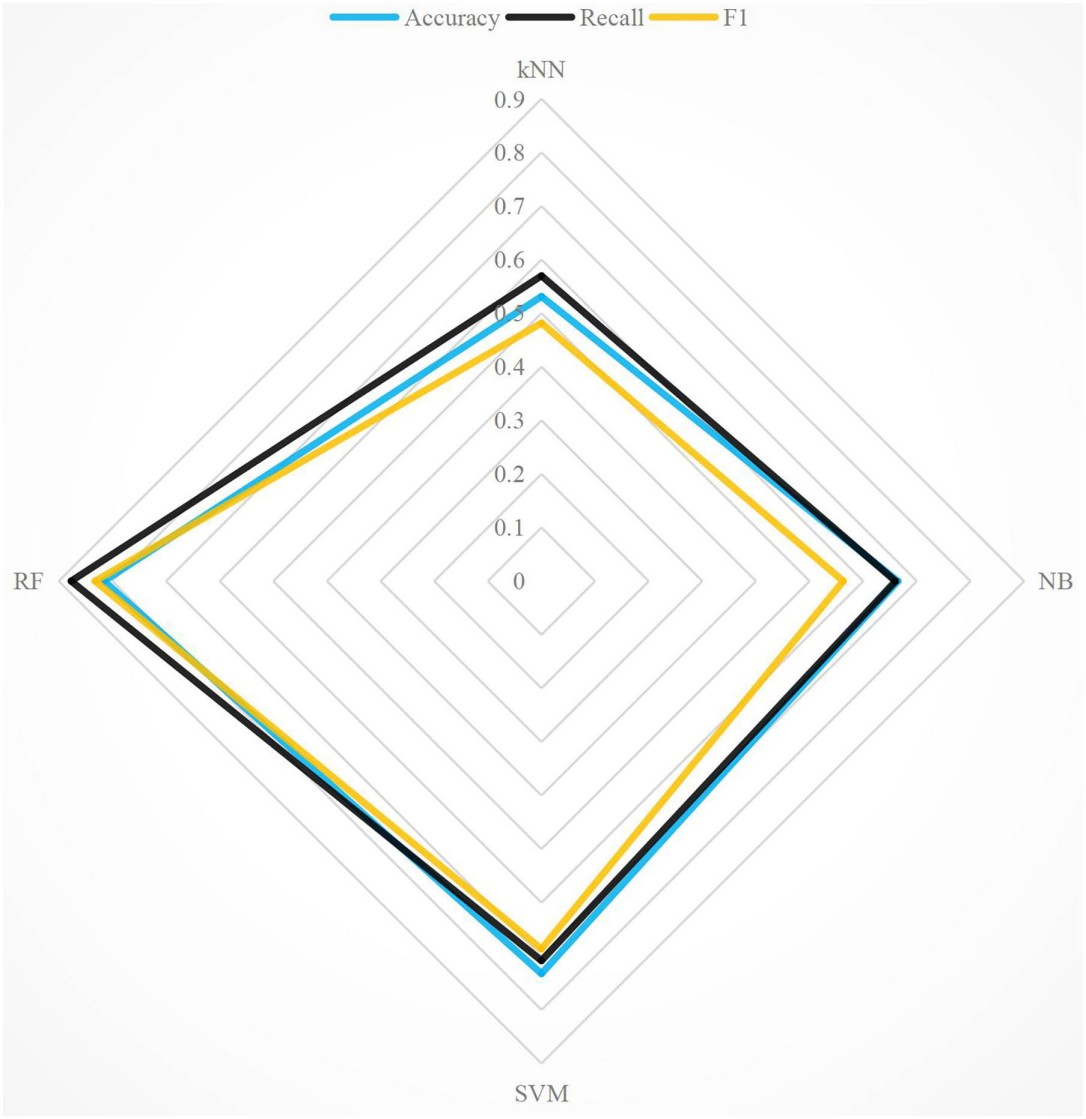
Comparison of evaluation metrics with four methods under extended corpora.

To analyze the emotional expression in literary works, this paper selected “Modern Fiction” to verify the emotional classification algorithm and used accuracy, recall, and F1 for evaluation metrics. “Modern Fiction” is an essay by Virginia Woolf, published in 1919. The reason for selecting “Modern Fiction” is that Virginia Woolf, known as a critic, did not analyze Russian and British literary works from an analytical perspective due to the influence of impressionism at that time. In “Modern Fiction,” Woolf argued that art's purpose is to expose people's imagination, knowledge, and experience of life, rather than to present a realistic “replica” of life. RF, support vector machine (SVM), k nearest neighbor (kNN), and naive Bayes (NB) are used to analyze the emotion of modern literary works (Bandhakavi et al., 2017). For the sake of intuition, the experimental comparison results of the four methods are compared in detail. The experimental comparison results of the four algorithms are shown in Figure 2. In Figure 2, the accuracy of RF is 9.86% higher than that of SVM and 46.77% higher than that of KNN. The recall of RF is 34.86% higher than that of NB and 49.86% higher than that of KNN. F1 of RF is 14.53% higher than that of SVM, 22.05% higher than that of NB, and 13.51% higher than that of KNN. Among the three evaluation metrics, F1 effectively integrates accuracy and recall, and F1 is the most appropriate evaluation metrics. Figure 3 shows the accuracy, recall, and F1 of the four algorithms under the extended corpus. It can be seen that the accuracy of emotion classification of literary works using RF is still very high, as well as recall and F1. The F1 of RF is the highest, so RF is used as the classification algorithm in this paper.

## Discussion

In this paper, we analyzed the emotion expression in the appreciation process of modern literary works, focused on the emotion analysis of figures depicted in literary works, and verified the accuracy of emotion classification through the machine learning algorithm RF of artificial intelligence. There are figures in literature whose emotions are so normal that they do not even feel their ups and downs. In the creation of literary works, environmental rendering can also be strengthened. Simply put, emotion expression should first work on the sense of painting. Compared with film and television works, literary works have another advantage in the expression of the figure's emotions, that is, psychological description. Film and television work mainly through sound and paintings to express the emotions of the figures. In literary works, the psychological state of the figures can be directly outlined in the word, and the thoughts of the figures can be underlined in words very frankly so that the readers can understand the inner feelings of the figures more easily. Some literary works have neither paintings nor psychological description of figures in scene processing. These kinds of superficial processing techniques are not easy to produce a sense of substitution, so readers cannot step into the inner world of the figures.

In addition to the emotion expression in the appreciation of modern literary works based on figure's emotion analysis mentioned in the paper, there is no shortcut to improve the ability of literary appreciation. It is a long process accumulated over a long period of time. We can also read the works repeatedly to cultivate language rhythm. In addition, we have to taste the language, gain aesthetic feeling, feel the connotations of modern literature, and imagine the situations described by the writers. However, at any rate, the appreciation of modern literary works is a process of perception, appreciation, and taste of beauty, and the result is that we must make appropriate criticism of literary works. We should resonate with the writers, which is beneficial to life taste and artistic accomplishment.

## Data Availability Statement

The raw data supporting the conclusions of this article will be made available by the author, without undue reservation.

## Ethics Statement

The studies involving human participants were reviewed and approved by the Ethics Committee of both Shanxi University and Shanxi Vocational University of Engineering Science and Technology. Written informed consent from the participants was not required to participate in this study in accordance with the national legislation and the institutional requirements.

## Author Contributions

JL contributed to methodology, writing, and result analysis.

## Conflict of Interest

The author declares that the research was conducted in the absence of any commercial or financial relationships that could be construed as a potential conflict of interest.

## Publisher's Note

All claims expressed in this article are solely those of the authors and do not necessarily represent those of their affiliated organizations, or those of the publisher, the editors and the reviewers. Any product that may be evaluated in this article, or claim that may be made by its manufacturer, is not guaranteed or endorsed by the publisher.
